# Effect of postconditioning on dynamic expression of tenascin-C and left ventricular remodeling after myocardial ischemia and reperfusion

**DOI:** 10.1186/s13550-015-0100-8

**Published:** 2015-04-02

**Authors:** Junichi Taki, Anri Inaki, Hiroshi Wakabayashi, Ichiro Matsunari, Kyoko Imanaka-Yoshida, Kazuma Ogawa, Michiaki Hiroe, Kazuhiro Shiba, Toshimichi Yoshida, Seigo Kinuya

**Affiliations:** Department of Nuclear Medicine, Kanazawa University Hospital, 13-1 Takara-machi, Kanazawa, 920-8641 Japan; The Medical and Pharmacological Research Center Foundation, Wo 32, Inoyama, Hakui, 925-0613 Japan; Department of Pathology and Matrix Biology, Mie University School of Medicine, 2-174 Edobashi, Tsu, 514-8507 Japan; Division of Pharmaceutical Sciences, Graduate School of Medical Sciences, Kanazawa University, Kakuma-machi, Kanazawa, 920-1192 Japan; Department of Nephrology and Cardiology, National Center for Global Health and Medicine, 1-21-1 Toyama, Shinjuku-ku, Tokyo, 162-8655 Japan; Division of Tracer Kinetics, Advanced Science Research Center, Kanazawa University, 13-1 Takara-machi, Kanazawa, 920-8640 Japan

**Keywords:** Tenascin-C, I-125-anti-tenascin-C antibody, Postconditioning, Ventricular remodeling, Myocardial ischemia and reperfusion

## Abstract

**Background:**

Tenascin-C (TNC), an extracellular matrix glycoprotein, is expressed transiently in distinct areas in association with active tissue remodeling. This study aimed to explore how ischemic postconditioning (PC) affects myocardial expression of TNC and ventricular remodeling using ^125^I-labeled anti-TNC antibody (^125^I-TNC-Ab) in a rat model of ischemia and reperfusion.

**Methods:**

In control rats (*n* = 27), the left coronary artery (LCA) was occluded for 30 min followed by reperfusion for 1, 3, 7, and 14 days. PC (*n* = 27) was performed just after the reperfusion. At the time of the study, ^125^I-TNC-Ab (1.0 to 2.5 MBq) was injected. Six to 9 h later, to verify the area at risk, ^99m^Tc-MIBI (100 to 200 MBq) was injected intravenously just after the LCA reocclusion, with the rats sacrificed 1 min later. Dual tracer autoradiography was performed to assess ^125^I-TNC-Ab uptake and area at risk. To examine the ventricular remodeling, echocardiography was performed 2 M after reperfusion in both groups.

**Results:**

In control rats, ^125^I-TNC-Ab uptake ratio at 1 day after reperfusion was 3.73 ± 0.71 and increased at 3 days (4.65 ± 0.87), followed by a significant reduction at 7 days (2.91 ± 0.55, *P* < 0.005 vs 3 days) and14 days (2.01 ± 0.17, *P* < 0.005 vs 1 and 3 days). PC attenuated the ^125^I-TNC-Ab uptake throughout the reperfusion time from 1 to 14 days; 2.59 ± 0.59 at 1 day, *P* < 0.05: 3.10 ± 0.42 at 3 days, *P* < 0.005: 1.93 ± 0.37 at 7 days, *P* < 0.05: 1.40 ± 0.07 at 14 days, *P* < 0.001. In echocardiography, PC reduced the ventricular end-diastolic and systolic dimensions (1.00 ± 0.06 cm to 0.83 ± 0.14 cm (*P* < 0.05) and 0.90 ± 0.15 cm to 0.62 ± 0.19 cm (*P* < 0.05), respectively) and prevented a decline of ventricular percentage fractional shortening (10.5 ± 3.7 to 28.2 ± 10.7, *P* < 0.005).

**Conclusions:**

These data indicate that ^125^I-TNC-Ab imaging may be a way to monitor myocardial injury, the subsequent repair process, and its response to novel therapeutic interventions like PC by visualizing TNC expression.

## Background

Extracellular matrix, in addition to cardiac cells, is essential to maintain the integrity of cardiac tissue and plays an important role not only in providing structural and mechanical support but also in modulating cell function. Cardiac repair after myocardial infarction is regulated through activation and suppression of the acute inflammatory process. Necrosis of cardiomyocytes provokes acute inflammation to remove dead cells and matrix debris, followed by timely infiltration of the infarcted myocardium with myofibroblasts. These secrete large amounts of extracellular matrix proteins, leading to replacement of myocardial dropout with mature collagen-based scar tissue in the infarct. These inflammatory and fibrotic processes are critically involved in the pathogenesis of ventricular remodeling, which is accompanied by changes in the structure and composition of the extracellular matrix and is a significant predictor of left ventricular dysfunction and an adverse prognosis [1].

Tenascin-C (TNC), an extracellular matrix glycoprotein, could provide important biological signaling that influences cell motility, proliferation, differentiation, survival, or apoptosis via cell-extracellular matrix interaction during tissue remodeling of various tissues [2,3]. TNC appears in the heart only in the early stages of embryonic development and is not normally expressed in the adult. However, it reappears transiently associated with myocardial injury such as myocardial infarction [4,5], hibernating myocardium [6], myocarditis [7-9], and dilated cardiomyopathy [10,11]. Its site-specific expression suggests that TNC plays important roles during tissue remodeling but also that it can serve as an indicator of myocardial disease activity.

Recently, other groups and we have reported increased serum TNC in heart failure patients, reflecting the severity and progression of ventricular remodeling [12-15]. Of particular interest is that serum levels of TNC within 1 week of infarction can be a possible predictor of ventricular remodeling and poor prognosis [16,17]. Also, our previous study implied that TNC expression might be altered if the ischemic insults were modified by cardioprotective interventions [18].

Postconditioning, defined as several repeated cycles of intermittent reperfusion and reocclusion after an index ischemia, is cardioprotective in a canine model of ischemia and reperfusion [19]. However, the effect of postconditioning on the TNC expression and left ventricular remodeling is unspecified. This prompted us to explore the effect of postconditioning on the spatial and temporal changes occurring in the expression of TNC after myocardial ischemia and reperfusion using ^125^I-labeled anti-tenascin-C antibody (^125^I-TNC-Ab) and left ventricular remodeling in a rat model of acute ischemia and reperfusion.

## Methods

### Animal model of acute ischemia and reperfusion

All experimental procedures involving animals were conducted in accordance with the institutional guidelines set by the Institute for Experimental Animals, Kanazawa University Advanced Science Research Center. Eight to 11-week-old male Wistar rats (*n* = 54) were anesthetized with an intraperitoneal administration of 40 mg of pentobarbital per kilogram and ventilated mechanically with room air. After left thoracotomy and exposure of the heart, a 7-0 polypropylene suture on a small curved needle was passed through the myocardium beneath the proximal portion of the left coronary artery (LCA), and both ends of the suture were passed through a small vinyl tube to make a snare. The suture material was pulled tight against the vinyl tube to occlude the LCA. Myocardial ischemia was confirmed by regional cyanosis of the myocardial surface and ST-segment elevation on the ECG. In control rats, LCA was occluded for 30 min and reperfusion was obtained by release of the snare and confirmed by the finding of a myocardial blush over the area at risk. In the group of rats with postconditioning, after the 30 min of LCA occlusion, 10 s of reperfusion followed by 10 s of LCA reocclusion was repeated six times at the beginning of the reperfusion. The snare was left loose on the surface of the heart for reocclusion of the LCA just before sacrifice to identify the area at risk (*18*). Groups of animals with and without postconditioning were studied at 1 (*n* = 5, 5), 3 (*n* = 6, 5), 7 (*n* = 4, 6), and 14 days (*n* = 6, 5, respectively) after reperfusion. At the time of the study, ^125^I-TNC-Ab (1.0 to 2.5 MBq) was administered via a tail vein. Six to 9 h afterward, 100 to 200 MBq of ^99m^Tc-MIBI was injected via a tail vein just after the reocclusion of the proximal portion of the LCA for delineation of the area at risk. One minute later, the rat was euthanized and the heart was removed for analysis. The excised heart was rinsed in saline, frozen in isopentane, cooled in dry ice, and embedded in methyl cellulose. Serial short-axis heart sections 20 μm thick were obtained by sectioning on a cryostat to create a series of rings for autoradiography.

To evaluate the left ventricular remodeling 2 months after reperfusion, 30 min of LCA occlusion followed by reperfusion with (*n* = 6) and without postconditioning (*n* = 6) was performed. Two months later, echocardiography was performed to evaluate the left ventricular dimensions and function. After echocardiography, 100 to 200 MBq of ^99m^Tc-MIBI was injected via a tail vein. One minute later, the rat was euthanized and the heart was removed for autoradiography.

### Radiolabeling of anti-tenascin-C antibody

Anti-TNC mouse IgG Fab’, a mouse monoclonal antibody against TNC, clone 4F10TT, was raised by immunization of a TNC-null mouse with purified human TNC as described previously [7].

Radiolabeling of anti-TNC antibody with ^125^I was achieved by the chloramine-T method [20]. Briefly, [^125^I]sodium iodide solution (37 MBq/10 μL, Perkin Elmer, Waltham, MA) was added to 60 μL of antibody in PBS (0.32 mg/mL). Following mixing, 4 μL of chloramine-T aqueous solution (1 mg/mL) was added. After 15 min at room temperature, the reaction was quenched with 20 μL of Na_2_H_2_SO_5_. The crude product was purified with a PD-10 column (GE Healthcare UK Ltd., Buckinghamshire, England) with saline as the eluate. The radiochemical purity of ^125^I-TNC-Ab was determined by thin layer chromatography (TLC). TLC analyses were performed with silica plates (Art 5553, Merck, Darmstadt, Germany) with saline as the developing solvent. Radiochemical purity was defined as the percent protein-bound activity, which was assessed by dividing the counts at the TLC origin by the total TLC counts. Specificity of the ^125^I-TNC-Ab was approved by the autoradiography with ^125^I-labeled nonspecific antibody in the previous study [18].

### Dual-tracer autoradiography

Dual tracer autoradiography of the left ventricular short axis slices was performed for the assessment of ^125^I-TNC-Ab uptake and ischemic area (or area at risk) demonstrated by ^99m^Tc-MIBI image. The first autoradiographic exposure on an imaging plate (BAS-MS, Fuji Film) was performed for 15 to 20 min to visualize the area at risk expressed by ^99m^Tc-MIBI distribution at 1 to 2 h after sacrifice. Three days later (12 half-lives of ^99m^Tc), the second exposure was made for 7 days to image the distribution of ^125^I-TNC-Ab.

### Data analysis

^125^I-TNC-Ab accumulation was evaluated in three myocardial slices at the mid-ventricular level spaced 1 mm apart. The distribution of the tracers was determined by analysis of the digitized autoradiographs. The photostimulated luminescence in each pixel (100 × 100 μm) was determined using a bioimaging analyzer (BAS-5000, Fuji Film). For quantitative analysis, the uptake values of each region of interest (ROI) were expressed as the background-corrected photostimulated luminescence per unit area (1 mm^2^). A background ROI was set adjacent to the left ventricle. Ischemic and normally perfused areas were defined from the ^99m^Tc-MIBI image, and these ROIs were applied to the ^125^I-TNC-Ab images to evaluate the uptake of ^125^I-TNC-Ab. ^125^I-TNC-Ab uptake area was also set manually as a ROI by circumscribing the increased activity of the ^125^I-TNC-Ab. ^125^I-TNC-Ab uptake ratio was calculated by dividing the uptake value in an ischemic area by that of a normally perfused area. The ratio of ^125^I-TNC-Ab uptake ROI area to an ischemic ROI area was defined as the percentage of the ^125^I-TNC-Ab uptake area. All parameters in each rat were expressed as the average value obtained from the analysis of three representative slices.

The autoradiography in rats just administered ^99m^Tc-MIBI after echocardiography 2 months after ischemia, and reperfusion was analyzed for myocardial viability. Area of infarction was defined arbitrarily with less than 60% of maximal ^99m^Tc-MIBI uptakes in the three representative slices.

### Immunohistochemical staining

Short axis frozen sections adjacent to the slices for autoradiography were mounted on slides. These short axial heart sections were washed with PBS and stained with mouse monoclonal anti-TNC mouse 4F10TT, the same antibody used for autoradiography, with direct immunoperoxidase technique. For paraffin sections, the heart was fixed in 4% paraformaldehyde, embedded in paraffin, and immunostained as previously described [5]. Briefly, after treatment with pepsin for 10 min for antigen retrieval, sections were incubated with anti-TNC antibody 4F10TT and subsequently with peroxidase-conjugated anti-mouse IgG Fab’ (Medical and Biological Lab Co. Ltd., Nagoya, Japan). After washing, diaminobenzidine/H_2_O_2_ solution was used to demonstrate antibody binding. The site and distribution of the expression of the TNC was examined with the aid of the hematoxylin-eosin stained slice adjacent to the immunohistochemical stained slice.

### Echocardiography

Echocardiography was performed in rats with (*n* = 6) and without (*n* = 6) postconditioning at 2 months after ischemia and reperfusion under anesthesia with 1% to 2% isoflurane using Vivid 7 (GE Healthcare, Milwaukee, WI, USA) with a 14-MHz i13L linear array transducer. Three cardiac cycles of the parasternal short axis view at papillary muscle level were recorded at a frame rate of 265 per second. The images were processed offline using commercially available software (EchoPac 6.1, GE Healthcare, Milwaukee, WI, USA). The left ventricular internal dimensions at the end of diastole (LVEDD) and at the end of systole (LVESD) were measured digitally using anatomical M-mode feature. LV fractional shortening (%FS) was calculated as [(LVEDD–LVESD)/LVEDD] × 100.

### Statistical analysis

All results were expressed as mean ± 1 SD. Statistical analyses were performed using a Macintosh computer with software JMP 5.0.1 J. Group comparisons were performed using Tukey-Kramer method to identify differences among groups. A value of *P* < 0.05 was considered statistically significant.

## Results

### Radiolabeling

^125^I-labeled anti-TNC antibody was prepared with high radiochemical yield (89%). After purification by PD-10, ^125^I-labeled anti-TNC antibody showed a radiochemical purity of over 99%.

### Size of area with ^125^I-TNC-Ab uptake against area at risk

**Figure 1:**
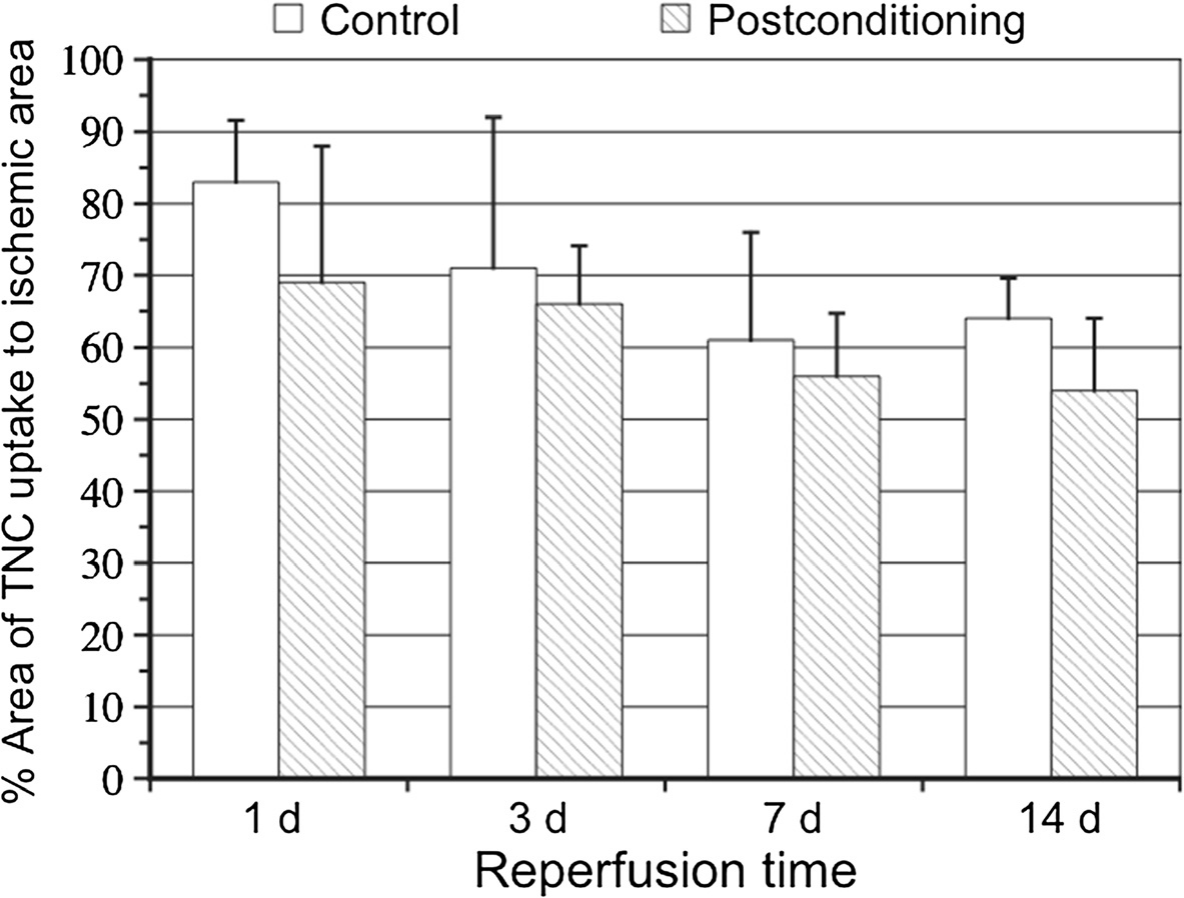
**Time course of the percentages of** ^**125**^ **I-TNC-Ab uptake area against area at risk (percentage area).** The percentages of ^125^I-TNC-Ab uptake areas without postconditioning at 1, 3, 7, and 14 days after reperfusion tended to decline over time but the differences were statistically insignificant (82.7% ± 8.6%, 70.6% ± 20.5%, 61.1% ± 15.1%, 63.8% ± 5.6%, respectively, *P* = ns). Postconditioning reduced the percentages of ^125^I-TNC-Ab uptake areas at all reperfusion times (at 1, 3, 7, and 14 days after reperfusion were 69.1% ± 6.6%, 65.9% ± 6.1%, 55.7% ± 6.5%, and 54.4% ± 3.5%, respectively) but not statistically significantly so (*P* = ns, vs without postconditioning).

The percentages of ^125^I-TNC-Ab uptake areas against area at risk in rats without postconditioning at 1, 3, 7, and 14 days after reperfusion tended to decline over time (82.7% ± 8.6%, 70.6% ± 20.5%, 61.1% ± 15.1%, 63.8% ± 5.6%, respectively, *P* = ns) (Figure 1).

Postconditioning reduced the percentages of ^125^I-TNC-Ab uptake areas against area at risk at all reperfusion times (values at 1, 3, 7, and 14 days after reperfusion were 69.1% ± 6.6%, 65.9% ± 6.1%, 55.7% ± 6.5%, and 54.4% ± 3.5%, respectively), with these differences not statistically significant (*P* = ns, vs without postconditioning) (Figure 1).

### ^125^I-TNC-Ab Uptake

**Figure 2:**
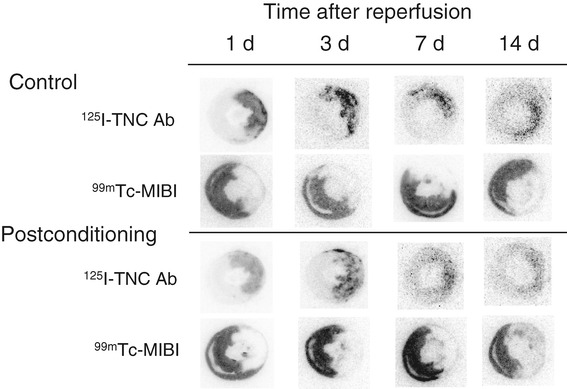
**Autoradiographies of** ^**125**^ **I-TNC-Ab and** ^**99m**^ **Tc-MIBI in rats with and without postconditioning.** Intense ^125^I-TNC-Ab uptake was observed in the area at risk that was represented by the perfusion defect of ^99m^Tc-MIBI at 1 and 3 days after reperfusion, followed by moderate uptake at 7 and 14 days after reperfusion. Postconditioning attenuated the ^125^I-TNC-Ab uptake at all reperfusion times.

In visual analysis, strong ^125^I-TNC-Ab uptake was observed in the area at risk at 1 and 3 days after reperfusion, followed by moderate uptake at 7 and 14 days after reperfusion. Postconditioning attenuated the ^125^I-TNC-Ab uptake throughout the course of reperfusion time (Figure 2).

In the quantitative analysis, ^125^I-TNC-Ab uptake ratio of the rats without postconditioning at 1 day after reperfusion was 3.73 ± 0.71 and increased at 3 days (4.65 ± 0.87), followed by a significant reduction at 7 days after reperfusion (2.91 ± 0.55, *P* < 0.005 vs 3 days) and followed by further reduction at 14 days (2.01 ± 0.17, *P* < 0.005 vs 1 and 3 days).

**Figure 3:**
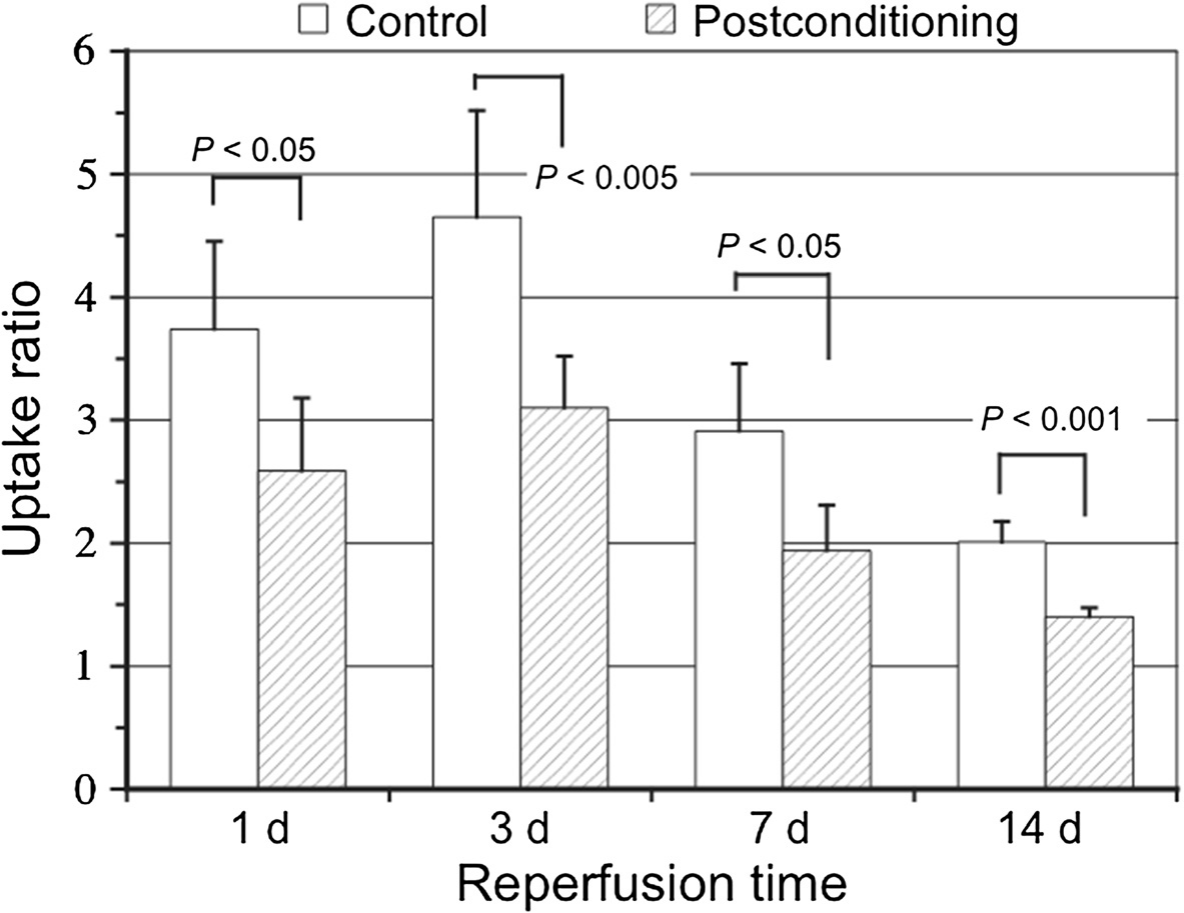
**Comparison between the** ^**125**^ **I-TNC-Ab uptake ratios in rats with and without postconditioning.** ^125^I-TNC-Ab uptake ratio was calculated by dividing the ^125^I-TNC-Ab count density in the area at risk by that of non-ischemic area. ^125^I-TNC-Ab uptake ratio in rats without postconditioning at 1 day after reperfusion was 3.73 ± 0.71 and increased at 3 days (4.65 ± 0.87), followed by a significant reduction at 7 days after reperfusion (2.91 ± 0.55, *P* < 0.005 vs 3 days) and a further reduction at 14 days (2.01 ± 0.17, *P* < 0.005 vs 1 and 3 days). Postconditioning attenuated the ^125^I-TNC-Ab uptake ratio throughout the reperfusion time from 1 to 14 days; 2.59 ± 0.59 at 1 day, *P* < 0.05: 3.10 ± 0.42 at 3 days, *P* < 0.005: 1.93 ± 0.37 at 7 days, *P* < 0.05, 1.40 ± 0.07 at 14 days, *P* < 0.001.

Postconditioning reduced the ^125^I-TNC-Ab uptake ratio throughout the time from 1 to 14 days after reperfusion; 2.59 ± 0.59 at 1 day, *P* < 0.05: 3.10 ± 0.42 at 3 days, *P* < 0.005: 1.93 ± 0.37 at 7 days, *P* < 0.05, 1.40 ± 0.07 at 14 days, *P* < 0.001 (Figure 3).

### Histopathological findings

**Figure 4:**
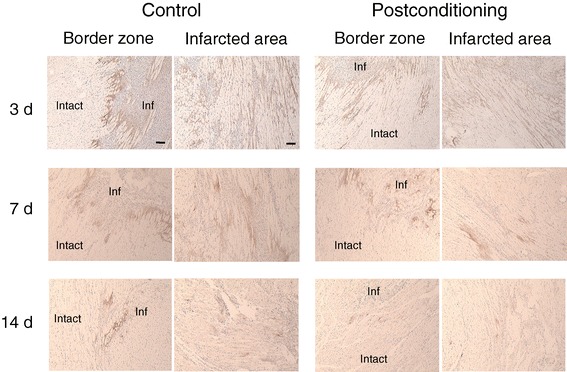
**Immunostaining for TNC at 3, 7, and 14 days after reperfusion with and without postconditioning is presented.** Strong TNC brown staining was seen in border zone between intact and infarcted area, and also scattered brown staining was seen throughout the infarcted area at 3 days after reperfusion. With postconditioning, TNC staining tended to be weaker in both border zone and infarcted area. At 7 and 14 days after reperfusion, TNC staining became weaker at both border zone and infarcted area. With postconditioning, TNC staining at both border zone and infarcted area became weaker. Intact, intact area; Inf, infarcted area; Scale bars, 100 μm.

At 1 to 3 days after ischemia and reperfusion, muscle lysis, muscle destruction, and coagulation necrosis of cardiomyocytes with severe inflammatory cell infiltration were observed in the infarcted area. TNC deposition was observed throughout the infarcted area with inflammation, though strong staining was limited to the border zone between infracted and intact areas. No TNC expression was detected in the intact area. This TNC expression pattern was not changed significantly by postconditioning, but staining of TNC tended to be weaker in both border zone and infarcted area. Then, necrotic tissue was gradually replaced by granulation tissue with attenuation of inflammation. TNC deposition gradually decreased until day 14. With postconditioning, the spatiotemporal pattern of TNC deposition came to resemble that of control animals, while the degree of staining became weaker (Figure 4).

### Left ventricular remodeling at 2 months after reperfusion

**Figure 5:**
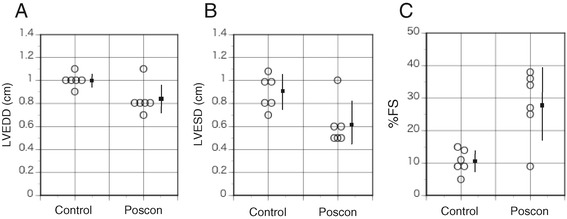
**Echocardiographic data 2 months after reperfusion in rats with and without postconditioning.** Compared with control rats, postconditioning significantly reduced the LVEDD **(A)** (*P* < 0.05), LVESV **(B)** (*P* < 0.05), and improved %FS **(C)** (*P* < 0.005). Small squares indicate the mean values, and bars show standard deviation. LVEDD, left ventricular diastolic dimension; LVESD, left ventricular systolic dimension; %FS, percentage of fractional shortening; Poscon, postconditioning.

Echocardiographic data at 2 months after ischemia and reperfusion are given in Figure 4. Heart rate in control rats and postconditioning was similar (389 ± 8.7/min and 401 ± 32/min, respectively, *P* = 0.40). Postconditioning reduced the LVEDD (1.00 ± 0.063 cm to 0.83 ± 0.14 cm, *P* < 0.05) (Figure 5a) and LVESD (0.90 ± 0.15 cm to 0.62 ± 0.19 cm, *P* < 0.05) (Figure 5b). Postconditioning also mitigated the left ventricular dysfunction (%FS; 10.5% ± 3.7% to 28.2% ± 10.7%, *P* < 0.005) (Figure 5c). Infarct size (percentage of left ventricle) by autoradiography in rats with postconditioning was significantly smaller than those without postconditioning (44.5% ± 7.9% vs 59.7% ± 9.0%, *P* < 0.05).

## Discussion

The present study revealed that ischemic postconditioning significantly attenuated TNC expression as demonstrated by the accumulation of ^125^I-TNC-Ab throughout the entire period until 2 weeks after ischemia and reperfusion.

Postconditioning also attenuated left ventricular remodeling and reduced infarct size at 2 months after ischemia and reperfusion. These data suggest that attenuation of TNC expression during the acute to subacute phase of tissue healing after infarction by postconditioning might be related to the attenuation of ventricular remodeling at 2 months after myocardial infarction, although whether the relationship is cause and effect or simple association remained to be elucidated.

Since the discovery of the cardio-protective effect of postconditioning defined as rapid sequential intermittent interruption of blood flow applied during early moments of reperfusion in a canine model of 60 min of myocardial ischemia and reperfusion [19], the cardio-protective effect induced by postconditioning has been extensively investigated and confirmed in many species, including man [21]. The attenuation of cardiac injury afforded by postconditioning has been attributed to suppression of reperfusion injury with consequent attenuation of myocardial apoptosis and necrosis. Many of the primary physiological endpoints of protection during reperfusion have been investigated including delay in washout of endogeneous autacoids, reduction of inflammatory response, protection of vascular endothelium, stimulation of survival kinase and transmitter, inhibition of death kinase, preservation of mitochondrial function, and reduction of cardiomyocyte loss [21]. Although a number of protective mechanisms targeted by postconditioning have been explored, it is generally recognized that inhibition of mitochondrial permeability transition pore opening by postconditioning is the final step in a complicated series of cellular events responsible for cell protection [21-23]. After 30-m ischemia of the isolated rat heart, mitochondrial permeability transition pore opening does not occur during ischemia but does within the first 5 m of reflow [24]. Therefore, it is important to induce postconditioning just after reperfusion to inhibit mitochondrial permeability transition pore opening, at which time its effect on the reduction of tissue damage has been demonstrated.

On the other hand, the effect of postconditioning on tissue repair including interstitial signaling and remodeling has been scarcely investigated. After myocardial infarction in the permanent ligation model, TNC is markedly but transiently upregulated during the early phase of tissue repair, is predominantly produced by interstitial fibroblasts in the vicinity of the injured cardiomyocytes but not by cardiomyocytes themselves [25], and is localized in the border zone between infarcted and viable myocardium [4,5] while in a reperfusion model it is scattered throughout the infarcted area in addition to border zone [18]. TNC may exert conflicting effects on cardiac tissue repair, healing, and ventricular remodeling [26,27]. TNC may loosen the strong adhesion of cardiomyocytes from the matrix and upregulate the expression and activity of matrix metalloproteinases [28,29], promoting degradation of the extracellular matrix and slippage of myocytes and facilitate inflammatory cell infiltration within the ventricular wall, resulting in an increased risk of ventricular thinning and dilatation. However, these functions promote cell rearrangement and allow myofibroblasts and capillary vessels to spread into tissue during the restoration process. Because of the limited regenerative ability of cardiomyocytes, the interstitial cells become a major player in myocardial tissue repair, with especially myofibroblasts playing an important role in wound healing by synthesizing collagens and exerting strong contractile forces to promote wound healing [30]. It is suggested that TNC produced by interstitial cells at an early phase of myocardial infarction promotes recruitment of myofibroblasts to injured sites by accelerating migration and differentiation and enhancing traction forces [25]. Therefore, these functions of TNC may facilitate tissue healing, reinforcement of the cardiac matrix, and fibrosis and prevent ventricular dilatation by generating traction forces [5,7,25].

Although it is difficult to conclude whether TNC is more beneficial or detrimental for tissue healing and reconstruction after myocardial infarction, a timely and proper degree of TNC expression might be beneficial for myocardial tissue repair, whereas prolonged overexpression of TNC might interfere with sound tissue repair and cause inappropriate reconstruction of infarcted ventricle, resulting in so-called ventricular remodeling.

In the mice model of permanent ligation of the coronary artery, ventricular remodeling in the TNC knockout mouse was significantly reduced and cardiac function was improved compared with the wild type at day 28 after myocardial infarction [31]. In patients with acute myocardial infarction, peak serum TNC level at 5 days after infarction is an important independent predictor of prognosis, and higher TNC level is associated with a greater risk of left ventricular remodeling at 6 months after infarction, indicating that overexpression of TNC may aggravate ventricular remodeling [16]. The results of the present study were consistent with those of these animal and human studies in that less TNC expression due to postconditioning was found to be associated with less ventricular remodeling.

The application of target radionuclide imaging of regional TNC expression as proposed in the present study holds the potential to quantify the extent, amount, and localization of TNC expression and relate the pathophysiological events to the tissue repair and remodeling occurring after severe myocardial ischemia. The current study also demonstrated that the ^125^I-TNC-Ab imaging approach after myocardial ischemia and reperfusion provides an opportunity to monitor the effect of postconditioning, potentially leading to novel therapeutic interventions, directed at the reducing the extracellular matrix remodeling occurring after myocardial infarction.

One of the limitations of this study was that the left ventricular function was assessed on only short axis slices in echocardiography, because remodeling and dilation mainly occur on the apical part of the ventricle after anterior myocardial infarction.

For future clinical application as a SPECT agent, ^123^I-TNC-Ab is required. Labeling with ^123^I can easily be performed in the same way as for ^125^I. *In vivo* SPECT imaging by ^111^In-TNC-Ab has already been obtained successfully at 6 h after administration of the tracer in a rat model of 3 days after permanent coronary artery ligation. The biodistribution study of ^111^In-TNC-Ab showed that target-to-blood, target-to-lung, and target-to-liver ratios were 3.3, 2.0, and 0.35, respectively [32]. On the other hand, our previous study demonstrated that rapid blood clearance and retention of ^125^I-TNC-Ab in the infarcted area, resulting in a decent target-to-blood ratio (1.85 ± 0.14 at 5 h and 4.8 ± 1.0 at 24 h after injection) and good target-to-lung ratio (1.97 ± 0.19 at 5 h and 4.9 ± 1.2 at 24 h after injection), indicate the feasibility of *in vivo* imaging at 5 h or later after tracer injection [18]. However, relatively high liver uptake resulted in a low target-to-liver ratio (0.66 at 5 h and 0.82 at 24 h), suggesting that careful interpretation might be required for myocardial uptake adjacent to the liver, although the ratio was improved compared to that of ^111^In-TNC-Ab.

## Conclusions

The present study demonstrated that postconditioning attenuates the whole process of TNC expression demonstrated by ^125^I-TNC-Ab after myocardial ischemia and reperfusion. Postconditioning also attenuated left ventricular remodeling at 2 months after ischemia and reperfusion. The obtained data also implied that radiolabeled TNC-Ab imaging may be useful as a noninvasive way to monitor the changes occurring during the process of myocardial injury and its repair by various therapeutic interventions after ischemia and reperfusion by visualizing TNC expression.

## Abbreviations

TNC: Tenascin-C
^125^I-TNC-Ab: ^125^I-labeled anti-tenascin-C antibody
LCA: Left coronary artery
TLC: Thin layer chromatography
ROI: Region of interest
LVEDD: Left ventricular internal dimensions at the end of diastole
LVESD: Left ventricular internal dimensions at the end of systole
%FS: LV fractional shortening
